# Mouse Y-Encoded Transcription Factor *Zfy2* Is Essential for Sperm Head Remodelling and Sperm Tail Development

**DOI:** 10.1371/journal.pone.0145398

**Published:** 2016-01-14

**Authors:** Nadege Vernet, Shantha K. Mahadevaiah, Fanny Decarpentrie, Guy Longepied, Dirk G. de Rooij, Paul S. Burgoyne, Michael J. Mitchell

**Affiliations:** 1 Division of Developmental Genetics, MRC National Institute for Medical Research, London, United Kingdom; 2 Department of Functional Genomics and Cancer, Institut de Génétique et de Biologie Moléculaire et Cellulaire, Illkirch Cedex, France; 3 The Francis Crick Institute, Mill Hill Laboratory, London, United Kingdom; 4 Reproductive Biology Group, Division of Developmental Biology, Department of Biology, Faculty of Science, Utrecht University, Utrecht, The Netherlands; 5 Center for Reproductive Medicine, Amsterdam Medical Center, University of Amsterdam, 1105 AZ Amsterdam, The Netherlands; 6 Aix Marseille Université GMGF, Marseille, France; 7 Inserm, UMR_S 910, Marseille, France; University of Maryland School of Medicine, UNITED STATES

## Abstract

A previous study indicated that genetic information encoded on the mouse Y chromosome short arm (Yp) is required for efficient completion of the second meiotic division (that generates haploid round spermatids), restructuring of the sperm head, and development of the sperm tail. Using mouse models lacking a Y chromosome but with varying Yp gene complements provided by Yp chromosomal derivatives or transgenes, we recently identified the Y-encoded zinc finger transcription factors *Zfy1* and *Zfy2* as the Yp genes promoting the second meiotic division. Using the same mouse models we here show that *Zfy2* (but not *Zfy1*) contributes to the restructuring of the sperm head and is required for the development of the sperm tail. The preferential involvement of *Zfy2* is consistent with the presence of an additional strong spermatid-specific promotor that has been acquired by this gene. This is further supported by the fact that promotion of sperm morphogenesis is also seen in one of the two markedly Yp gene deficient models in which a Yp deletion has created a *Zfy2/1* fusion gene that is driven by the strong *Zfy2* spermatid-specific promotor, but encodes a protein almost identical to that encoded by *Zfy1*. Our results point to there being further genetic information on Yp that also has a role in restructuring the sperm head.

## Introduction

The mouse Y chromosome has two copies of a gene, *Zfy*, which together with the closely related X chromosome linked *Zfx*, encode zinc finger transcription factors [1–3]. They derive from an autosomal precursor that was added to the mammalian sex chromosomes via the pseudoautosomal region (PAR) subsequent to the separation of the marsupial and eutherian lineages [4, 5]. Postnatal expression of the mouse Y genes (*Zfy1* and *Zfy2*) is restricted to spermatogenic cells [6–8]. *Zfy2* encodes a much more potent transcription factor than *Zfy1* [9, 10] and has also acquired an additional strong spermatid-specific promoter [6] suggesting an important role during spermiogenesis. Although these mouse ‘*Zf*’ genes were identified in 1989–1990 [1, 2, 11] only recently have any spermatogenic functions been ascribed to them [12–14].

This study is a sequel to our two earlier papers documenting roles for mouse *Zfy* with respect to the apoptotic elimination of spermatocytes with univalent chromosomes at the first meiotic metaphase [13] and for the completion of the second meiotic division [14]. The latter paper has an extended introduction that describes the ‘raison d’être’ for the *Zfy* transgene additions to Yp gene deficient mice; this will be unfamiliar to most readers and can be accessed via the link http://www.ncbi.nlm.nih.gov/entrez/query.fcgi?cmd=Retrieve&db=PubMed&dopt=Citation&list_uids=24967676.

**Fig 1:**
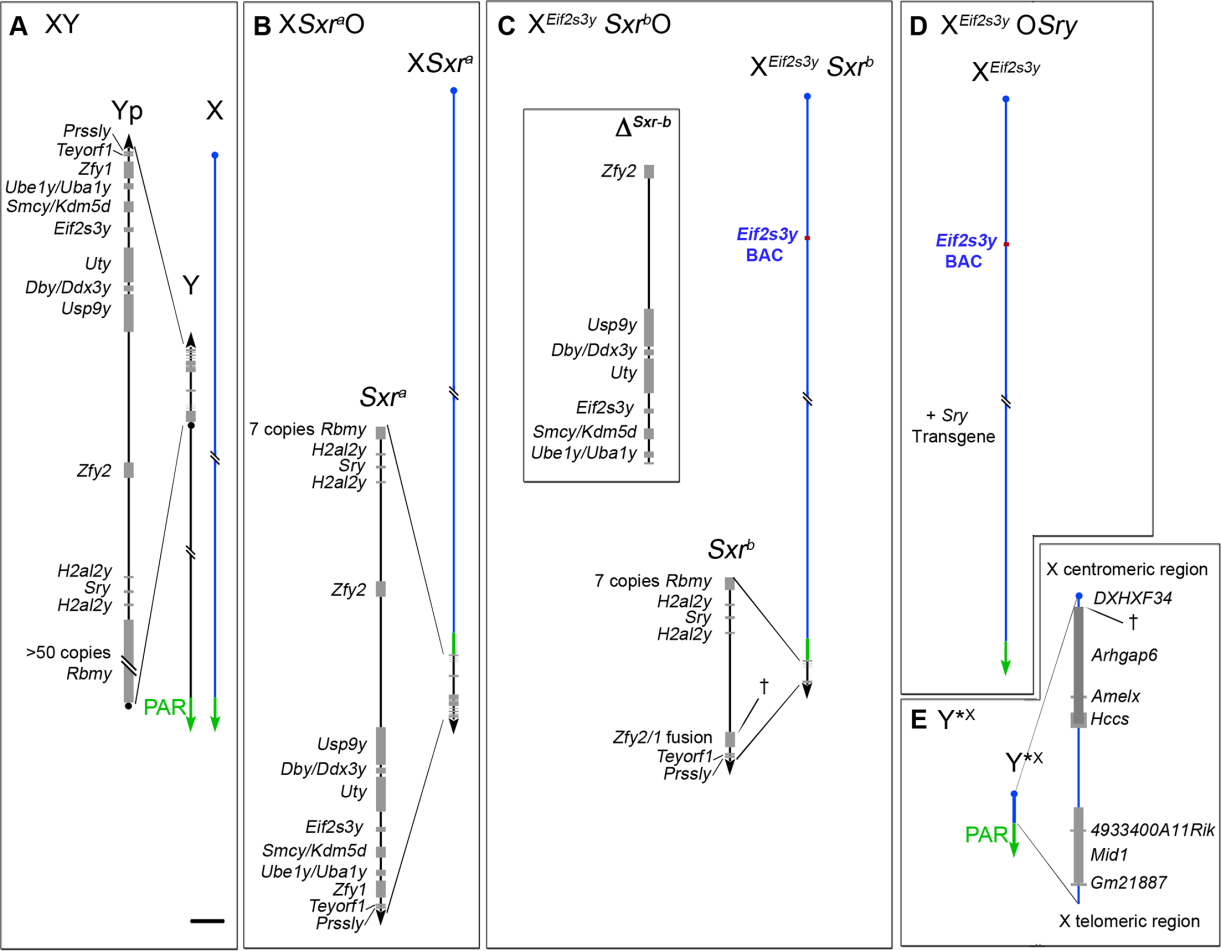
The XO and XY*^X^ mouse models. **(A)** XY. The Y short arm (Yp) gene complement of an XY male (represented to scale in the magnified view) comprises nine single copy genes, two duplicated genes and one multi copy gene. The pseudoautosomal region (PAR) located distally on the Y long arm mediates pairing and crossing over with the X PAR during meiosis to generate the XY sex bivalent. Centromeres are represented by a dot on the chromosome. **(B–D)** The diminishing Yp gene complements for the three XO male mouse models that lack the Y long arm. **(B)** X*Sxr*^*a*^O. The Yp-derived *Sxr*^*a*^ attached distal to the X PAR provides an almost complete Yp gene complement. **(C)** X^*Eif2s3y*^*Sxr*^*b*^O. The *Sxr*^*a*^-derived deletion variant *Sxr*^*b*^ has a 1.3 Mb deletion (Δ^*Sxr-b*^) removing 6 single copy genes and creating a *Zfy2/1* fusion gene spanning the deletion breakpoint (†). The deleted gene *Eif2s3y* is necessary for normal spermatogonial proliferation, so an X-located *Eif2s3y* transgene has been added. **(D)** X^*Eif2s3y*^OSry. This model has only two Yp genes—the testis determinant *Sry* provided as an autosomally located transgene and the spermatogonial proliferation factor *Eif2s3y* provided as the X-located transgene. E. Y*^X^. This mini sex-chromosome is an X chromosome with a deletion from just proximal to *Amelx* to within the DXHXF34 repeat adjacent to the X centromere († marks the deletion breakpoint). This X chromosome derivative has a complete PAR that can pair and crossover with the PAR of X*Sxr*^*a*^, X*Sxr*^*b*^ or X to form a ‘minimal sex bivalent’. Scale bar for magnified views is 150 kb.

In 2012 [15] we established that in X^*Eif2s3y*^O*Sry* males (Fig 1D) in which the only Yp genes present are an X located *Eif2s3y* transgene and an autosomally located *Sry* transgene, there is a block at step 7 of the round spermatid stage of sperm development, whereas in X*Sxr*^*a*^O and X^*Eif2s3y*^*Sxr*^*b*^O males (Fig 1B and 1C) there was evident sperm morphogenesis. *Sxr*^*a*^ provides a near complete Yp gene content, whereas *Sxr*^*b*^ has a very limited Yp gene content (Fig 1A–1C); however, *Sxr*^*b*^ is unique in having a *Zfy2/1* fusion gene that includes the strong spermatid specific promotor from *Zfy2*. This led us to hypothesize that this spermatid specific promotor is of paramount importance in enabling *Zfy* to provide an essential function during sperm morphogenesis—in normal males this function would primarily be provided by *Zfy2*. In the current study we show through *Zfy1* and *Zfy2* transgene additions to Yp gene deficient models that this is indeed the case.

## Results

For our published study showing that *Zfy1* and *Zfy2* have an important role in promoting completion of the second meiotic division to form haploid spermatids [14] we added a minute sex chromosome (Y*^X^) [16, 17]) to the three XO models. Y*^X^ (Fig 1E) comprises a complete pseudoautosomal region (PAR), a short ‘X telomeric region’, some repeated sequences mapping adjacent to the X centromere, and a presumed X-derived centromere [18–20]. Y*^X^ was previously shown to enable the formation of a sex bivalent, thus circumventing the MI SAC [21]. We abbreviate the three XY*^X^ models as XY*^X^*Sxr*^*a*^, X^*E*^Y*^X^*Sxr*^*b*^ and X^*E*^Y*^X^*Sry*; the X-located *Eif2s3y* transgene is denoted X^*E*^. Importantly in the context of the present study, we found that the addition of Y*^X^ had no effect on the frequency of haploid spermatids in the context of *Sry*, but it markedly increased the haploid frequency in the context of *Sxr*^*b*^ (Figure 3A in reference [14]). Therefore, before assessing the effects of *Zfy* transgene additions on sperm morphogenesis we sought to establish: (1) whether the Y*^X^ addition *per se* had any discernible effect on spermiogenic progression by comparing X^*E*^O*Sry* with X^*E*^Y*^X^*Sry*; and (2) whether the markedly increased haploid frequency associated with the Y*^X^ addition in the context of *Sxr*^*b*^ had any discernible effect on spermiogenic progression by comparing X^*E*^*Sxr*^*b*^O with X^*E*^Y*^X^*Sxr*^*b*^.

### The addition of Y*^X^ does not enhance spermatid elongation or the associated sperm morphogenesis

**Fig 2:**
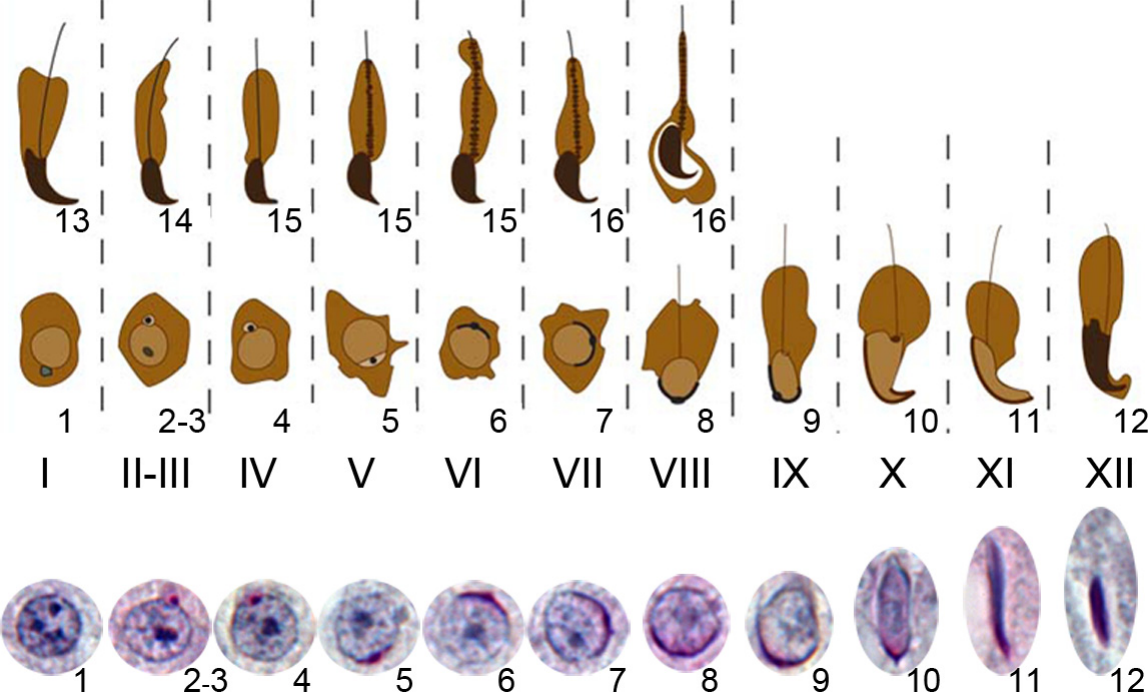
The normal steps of spermiogenesis in relation to testis tubule stages in an XY mouse. This figure presents a schematic depiction of the 16 steps of mouse spermiogenesis based on the stage of the spermatogenic cycle (stages I–XII). Photos of step 1–12 spermatid nuclei are shown with PAS staining at the foot of the diagram. The transition from round to elongating spermatids takes place during stage VIII; this marks the beginning of sperm morphogenesis. The shaping of the sperm head and formation of the sperm tail are essentially complete by step 12. However, the sperm are not shed until the following stages VIII-IX; consequently two generations of spermatids are present in stages I-VIII.

Fig 2 provides a summary diagram of spermiogenic progression in normal XY males based on the classical descriptions of the spermiogenic cycle in relation to testis tubule stage assessed from tubule sections. For the purposes of this study the key spermiogenic steps are 1–12 that are present in tubule stages I-XII. Haploid round spermatids first appear at stage I as a consequence of the two meiotic divisions occurring in the preceding stage XII. During stages I-VII the round spermatids form an acrosomal cap covering one side of the nucleus. During stages VIII-XII the main features of sperm morphogenesis take place (formation of the sperm tail; restructuring and condensation of the spermatid nucleus to form the sperm head). Spermatid development in all three XO models (Fig 1B and 1D) is aberrant and delayed to varying degrees [15], so assessment of tubule stage has to be based entirely on the spermatogonial and meiotic stages (see Materials and Methods).

**Fig 3:**
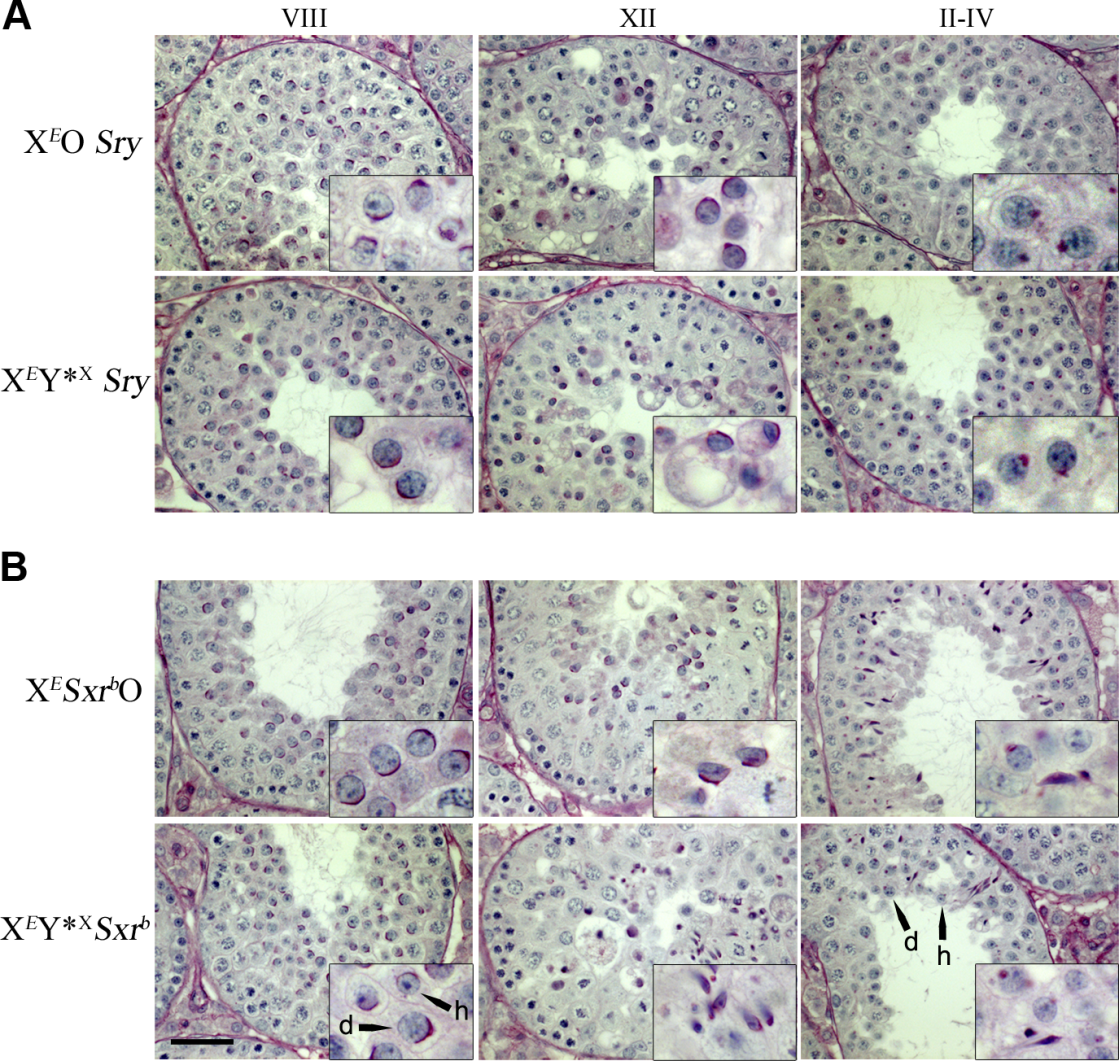
Addition of Y*^X^ to X^*E*^O*Sry* or X^*E*^*Sxr*^*b*^O models does not improve spermiogenic progression. Periodic acid Schiff/hematoxylin stained testis sections illustrating the extent of spermiogenic progression. Roman numerals denote estimated tubule stages. **(A)** In X^*E*^O*Sry*, the predominantly diploid spermatids do not elongate and the acrosomes (stained dark pink) remain randomly orientated relative to the basement membrane of the tubule (insets in VIII and XII). The spermatid nuclei show signs of pycnosis by stage XII (inset) and the cells have been eliminated by stages II-IV. The abundant round cells at stages II-IV are the new generation of round spermatids with early stages of acrosome development (dark pink ‘acrosomal granules’ in inset) [for more details see Vernet et al 2012]. In X^*E*^Y*^X^*Sry* the block to spermiogenesis remains with elimination of the arrested cells once again evident by stages II-IV (see inset). **(B)** In X^*E*^*Sxr*^*b*^O, at stage VIII the spermatids have not elongated and they are randomly orientated relative to the tubule basement membrane. However, as previously reported (Vernet et al 2012), spermatid elongation is delayed rather than absent, and is apparent by stage XII. Nuclear condensation is also delayed as it is not evident at stage XII, but many of the elongating spermatids survive to stages II-IV at which point nuclear condensation is now evident. In X^*E*^Y*^X^*Sxr*^*b*^ spermatid elongation and nuclear condensation is similarly delayed, but there appear to be fewer elongating spermatids surviving to stages II-IV [note the now evident haploid (h) as well as diploid (d) spermatids]. Scale bar is 40 μm (insets are x3 magnification).

The spermiogenic progression in X^*E*^Y*^X^*Sry* males proved to be equivalent to that in X^*E*^O*Sry* males (Fig 3A). In epithelial stage VIII the spermatids fail to re-orientate the acrosome to face the basal membrane, which normally precedes spermatid elongation. Moreover, the spermatids do not elongate and remodel the chromatin to form the sperm head (even by stage XII), and in both models the arrested, still round, spermatids are eliminated by stages II-IV of the following cycle. Thus the Y*^X^ addition *per se* has no effect on spermiogenic progression.

In agreement with our previous findings, X^*E*^*Sxr*^*b*^O males differ markedly from X^*E*^O*Sry* males (Fig 3B). In X^*E*^*Sxr*^*b*^O males spermatids do re-orientate such that the acrosomes come to face the basal membrane and spermatid elongation and subsequent nuclear condensation do take place. However, this spermiogenic progression is delayed relative to XY controls [15]; thus re-orientation does not occur during stage VIII, and elongation and some shaping of the sperm head with associated acrosome relocation are only evident by stage XII instead of stage IX. Correctly orientated spermatids with condensed nuclei are apparent by stages II-IV instead of X-XI. A similar pattern of spermiogenic progression can be seen in X^*E*^Y*^X^*Sxr*^*b*^ males (Fig 3B) with elongation and condensation of the spermatid nuclei and the correct orientation of elongating spermatids being most obvious in stage XII tubules and beyond.

### Addition of *Zfy2* (but not *Zfy1*) to X^*E*^Y*^X^*Sry* males promotes substantial sperm morphogenesis

**Fig 4:**
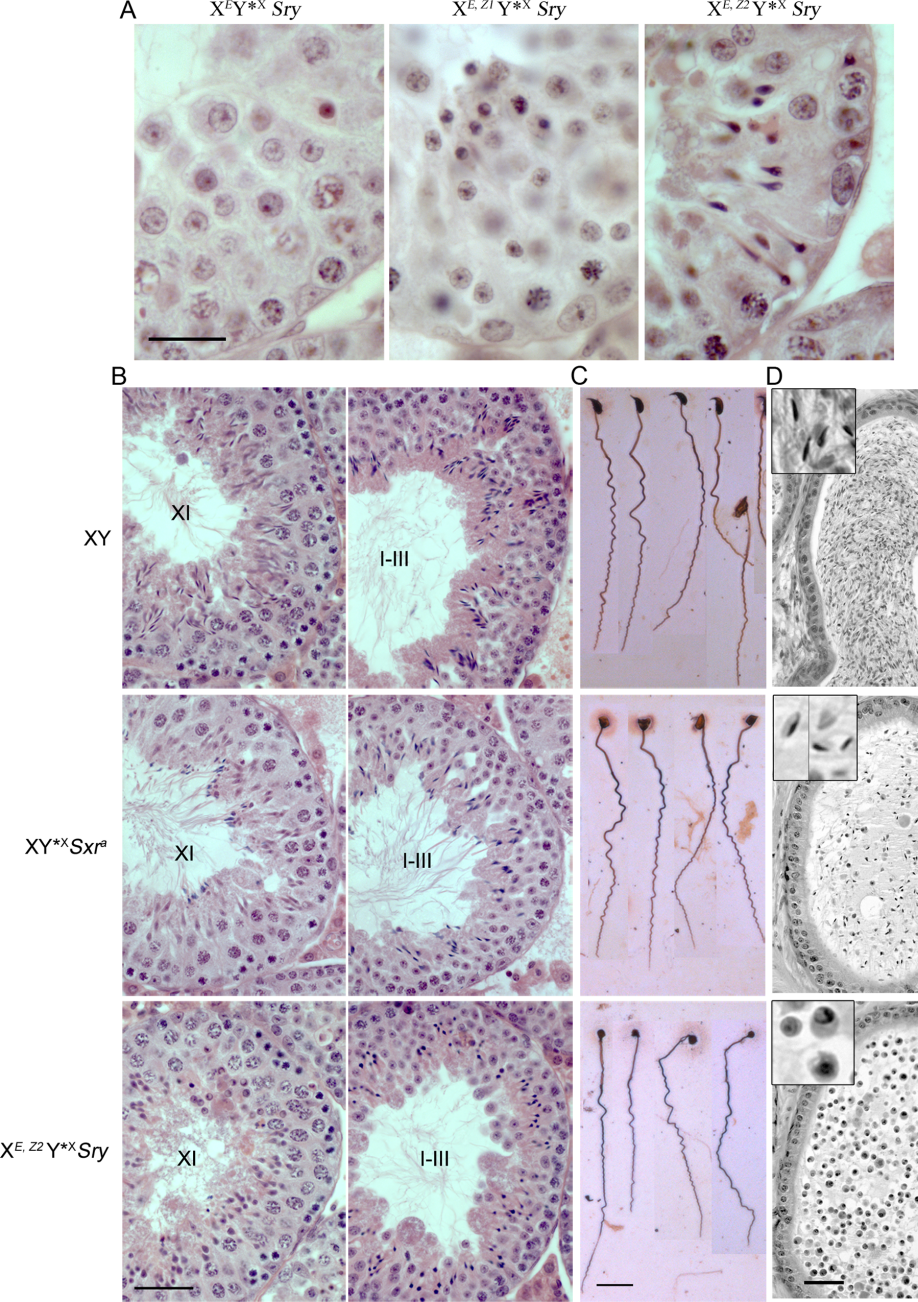
Addition of *Zfy2* (but not *Zfy1*) to X^*E*^Y*^X^*Sry* enables re-orientation of spermatids, together with clear head and tail morphogenesis; however the *Zfy2* transgene is not as effective as *Sxr*^*a*^ in supporting sperm morphogenesis. Hematoxylin and eosin (H & E) stained testis tubule sections. **(A)** By comparison with X^*E*^Y*^X^*Sry*, it is clear that the *Zfy1* transgene addition has no beneficial effect on spermiogenic progression whereas the *Zfy2* addition leads to the formation of correctly orientated elongated spermatids which have condensed sperm heads and some tail formation. **(B)** By comparison of X^*E*,*Z2*^Y*^X^*Sry* with XY*^X^*Sxr*^*a*^ (and XY controls) it is clear that *Sxr*^*a*^ is more effective than the *Zfy2* transgene in supporting the sperm morphogenesis. In agreement with the illustration in Fig 2, in XY males at stage XI sperm head morphogenesis has progressed to the early ‘hooked tip’ stage and nuclear condensation is evident. As previously reported, in XY*^X^*Sxr*^*a*^ at this stage some of the sperm from the previous cycle have not yet been shed (stained dark blue); the spermatids from the new cycle are retarded with respect to elongation and nuclear condensation is not evident. In X^*E*,*Z2*^Y*^X^*Sry* spermatid elongation is further retarded. Nevertheless, by stage I-III many spermatids with condensed chromatin are found in XY, XY*^X^*Sxr*^*a*^ and X^*E*,*Z2*^Y*^X^*Sry*. **(C)** Images of testicular sperm found in silver stained testicular cell smears. All three genotypes proved to have some sperm present in testicular cell smears. In XY*^X^*Sxr*^*a*^, as previously reported for epididymal sperm and testicular sperm, the sperm heads rarely have a hooked tip. In X^*E*,*Z2*^Y*^X^*Sry* the developing sperm heads show limited elongation. In all three genotypes the tails were well developed. **(D)** Hematoxylin/eosin stained epididymal tubule sections. In XY there are abundant sperm present with the heads showing the characteristic hooked tip. In XY*^X^*Sxr*^*a*^ the sperm heads rarely have a hooked tip. In X^*E*,*Z2*^Y*^X^*Sry* males no sperm could be identified; instead degenerating round spermatids are found suggesting that there has been some shedding of round spermatid stages despite the addition of *Zfy2*. Scale bars: A = 30 μm, B = 40 μm, C = 20 μm, D = 100 μm. Insets in D = 3x magnifications.

The effects of the *Zfy1* or *Zfy2* transgene additions were assessed in X^*E*^Y*^X^*Sry* males in which the only Y genes present are *Sry* and the X-located *Eif2s3y*; the *Zfy* transgenes used were single copy and also located on the X chromosome, and we denote the resulting males as X^*E*,*Z1*^Y*^X^*Sry* and X^*E*,*Z2*^Y*^X^*Sry*. In agreement with our expectation, addition of *Zfy1* had no discernible effect on spermiogenic progression, with the spermatids failing to re-orientate or elongate. In marked contrast, the *Zfy2* addition did promote spermiogenic progression (Fig 4A). Indeed, tubule sections of X^*E*,*Z2*^Y*^X^*Sry* males in stages X to VII all showed elongating spermatids with condensed nuclei and a tail being formed.

We next wished to assess whether the *Zfy2* addition reinstated spermiogenic progression to the same extent as that supported by the near complete Yp gene complement present in *Sxr*^*a*^. For this we compared spermiogenesis in X^*E*,*Z2*^Y*^X^*Sry* males with that in XY*^X^*Sxr*^*a*^ males. The latter have *Zfy1* and *Zfy2* encoded within *Sxr*^*a*^ but, like X^*E*,*Z2*^Y*^X^*Sry* males, lack genetic information encoded by the mouse Y long arm that is important for sperm head shaping [22–25]. We also included wild type XY controls in order to check at what tubule stage the abnormal sperm head shaping in XY*^X^*Sxr*^*a*^ became evident. Detailed analysis of staged seminiferous epithelium revealed spermiogenic anomalies in X^*E*,*Z2*^Y*^X^*Sry* males relative to both XY*^X^*Sxr*^*a*^ and wild type control genotypes (Fig 4B). At stage XI, there was a marked delay in spermatid elongation and nuclear condensation, and by stages I-III the spermatids looked unhealthy with darkly stained nuclei. Silver stained testicular cell smears showed that these abnormal spermatids were nevertheless capable of developing tails (Fig 4C). We then examined epididymal tubule sections in order to assess the relative numbers of testicular sperm passing from the testis into the epididymis (Fig 4D). As previously established [21], in XY*^X^*Sxr*^*a*^ males substantial numbers of sperm reach the epididymis although these sperm have abnormally shaped heads that rarely have a hooked tip. However, no sperm were seen in epididymal sections from X^*E*,*Z2*^Y*^X^*Sry* males. Instead, there were many round spermatids, indicating that substantial numbers of these cells slough off from the seminiferous epithelium in the *Zfy2* transgenics.

**Fig 5:**
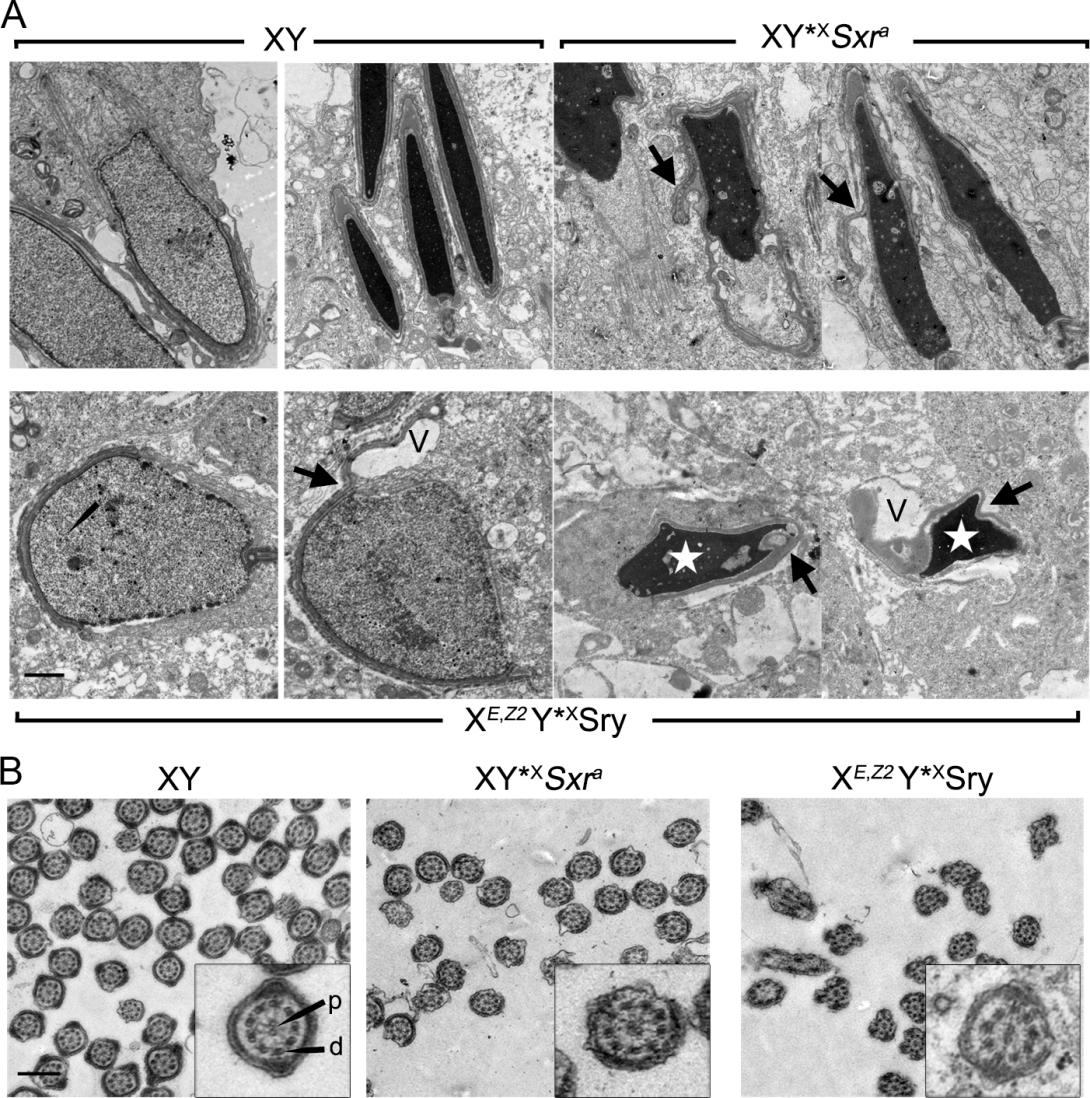
Sperm head and tail morphogenesis in X^*E*,*Z2*^Y*^X^*Sry* males as compared to controls. **(A)** Electron micrographs of developing sperm heads from 6 week old XY, XY*^X^*Sxr*^*a*^ and X^*E*,*Z2*^Y*^X^*Sry* males. Spermatids at round to elongating transitional stage in X^*E*,*Z2*^Y*^X^*Sry* present no apparent ultrastructural defects (bottom left picture). However, vacuoles (V) appear in the cytoplasm of elongating and condensing spermatids. Irregular spread of the acrosomal cap (arrows) distorting the spermatid nuclei is observed in X^*E*,*Z2*^Y*^X^*Sry* and XY*^X^*Sxr*^*a*^. It was again evident that the sperm heads in X^*E*,*Z2*^Y*^X^*Sry* fail to elongate properly (stars). **(B)** Electron micrographs of sperm tail sections showing a normal 9x2+2 axoneme pattern with a central microtubule pair (p) in addition to the nine outer doublets (d) in all three genotypes. Scale bars: A = 1 μm, B = 0.5 μm (Insets = 3x magnification).

Finally we assessed sperm morphogenesis by electron microscopy. Sperm heads from XY*^X^*Sxr*^*a*^ males elongate similarly to those of wild type males except for some acrosome invagination. In contrast, X^*E*,*Z2*^Y*^X^*Sry* males have compromised sperm head morphogenesis with signs of necrosis, acrosome invagination, incomplete elongation, and formation of cytoplasmic vacuoles (Fig 5A). Degenerating condensed spermatids were often present in large cytoplasmic inclusions within the epithelium, indicative of removal by apoptosis (not shown). However, cross sections of developing sperm tails appeared grossly normal with the classic axoneme structure composed of a central microtubule pair and nine outer doublets (9x2+2) (Fig 5B).

Clearly, although addition of the *Zfy2* transgene overcomes the step 7 round spermatid arrest to allow substantial sperm morphogenesis, qualitatively and quantitatively it does not match that achieved with the near complete Yp gene complement present in XY*^X^*Sxr*^*a*^ males.

## Discussion

The starting point for the present study was our finding that in X^*E*^O*Sry* males (Y gene complement *Eif2s3y* and *Sry*) there is a clear-cut and unique arrest of spermiogenesis at step 7 of spermatid development [15]. In the present study we showed that this arrest is also seen in X^*E*^Y*^X^*Sry* males that have the same Y gene complement, but also have the minute Y*^X^ chromosome that enables the formation of a sex bivalent, thus avoiding MI SAC responses. The transition from spermatid step 7 to step 8 is pivotal in spermiogenesis: at step 7 the spermatids are round and randomly oriented with respect to the basal membrane, whereas at step 8 the spermatids have re-orientated so that the developing acrosomal cap is now facing the tubule periphery. This re-orientation heralds the dramatic restructuring of the round spermatids to form spermatozoa (‘sperm morphogenesis’). We hypothesized that the arrest at step 7 was a consequence of a lack of *Zfy2*-dependent transcriptional changes, and that the expression of *Zfy2* at the critical stage was dependent on the alternative, strong, *Cypt*-derived, spermatid-specific promotor acquired by *Zfy2* during the evolution of mice (muridae) [15]. Our present results clearly demonstrate that *Zfy2* supports sperm morphogenesis, while *Zfy1*, which lacks this promotor, does not.

The transcription factors encoded by *Zfy1* and *Zfy2*, together with their X-linked homologue *Zfx*, are predicted to bind the same DNA sequence [26–28]. In most eutherian mammals *Zfx* and *Zfy* are widely expressed, and *Zfx* is exempt from X dosage compensation, suggesting a constraining dosage requirement in somatic tissues [29, 30]. However, in the myomorph rodent lineage, *Zfx* became subject to X-dosage compensation and the *Zfy*-encoded proteins diverged [1, 29, 31], and mice (muridae) ended up with two copies of *Zfy* with postnatal transcription restricted to spermatogenic cells [6–8]. We have previously discussed the evolutionary pressures that may have led to ZFY2 having a more potent transactivation domain than ZFY1, and ZFY1 frequently lacking the transactivation domain as a consequence of alternative splicing [14]. Here we consider the acquisition of the additional *Cypt*-derived promotor in the muridae [6, 9]. It is clear from recent studies that the mouse Y chromosome gene complement has been markedly affected by a post-meiotic X-Y genomic conflict, with the round spermatid specific genes *Sly* and *Slx* being key protagonists [22, 32–36]. As a consequence of this conflict there has been co-amplification of *Sly* and *Slx* (50–100 copies located on the mouse Y long arm [35]) with *Sly* expression serving to damp down transcription of the X and Y chromosomes in spermatids [22]. Progressive reduction of *Zfx* and *Zfy* gene transcription in spermatids as the genomic conflict continued will have provided a strong selective force for the recruitment of the strong *Cypt*-derived spermatid-specific promotor to *Zfy2*, in order to maintain sperm morphogenesis.

**Fig 6:**
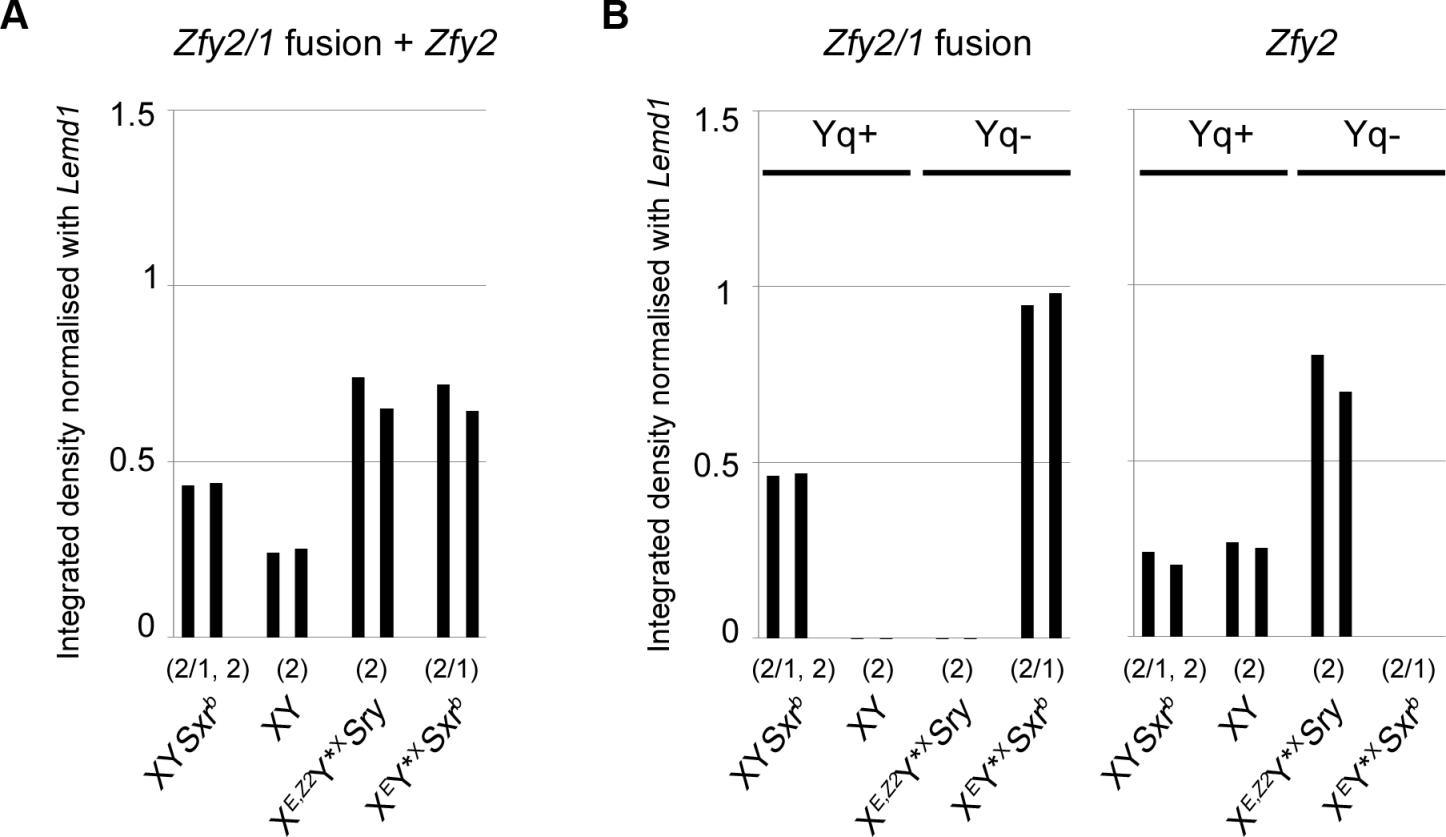
Levels of *Cypt*-dependent transcripts for XY*Sxr*^*b*^, XY, X^*E*,*Z2*^Y*^X^*Sry* and X^*E*^Y*^X^*Sxr*^*b*^. The RT-PCR bands quantified are those from the RT-PCR assay in S1 Fig. The transcripts expected for each genotype (n = 2 represented by two bars on the chart) are indicated above each genotype label (2/1 denotes *Cypt*-dependent *Zfy2/1* transcripts and 2 denotes *Cypt-*dependent *Zfy2* transcripts). **(A)** The PCR primers for this assay amplified *Zfy2/1* and *Zfy2* transcripts; only XY*Sxr*^*b*^ has both transcripts. It can be seen that the level of *Cypt-Zfy2/1* transcripts in X^*E*^Y*^X^*Sxr*^*b*^ is comparable to the level of *Cypt-Zfy2* transgene transcripts in X^*E*,*Z2*^Y*^X^*Sry*. **(B)** For the left panel the PCR primers are specific for *Zfy2/1*; the second and third genotypes lack the *Zfy2/1* fusion gene so the low level signal represents ‘background’. For the right panel the PCR primers are specific for *Zfy2*; in this case it is the fourth genotype that lacks *Zfy2*. The genotypes lacking the Y long arm (Yq-) have substantially higher transcript levels than the genotypes with a complete Y (Yq+).

Our original focus on the alternative *Cypt*-derived *Zfy2* promotor was triggered by our finding of substantial sperm morphogenesis in X^*Eif2s3y*^Y*^X^*Sxr*^*b*^ males in which a 1.3 Mb Yp deletion (Δ^*Sxrb*^–see Fig 1C) has created a *Zfy2*/1 fusion gene that includes the same promotor. We have developed a new PCR assay that specifically amplifies *Cypt* promotor dependent transcripts with exon 6 that encodes the transactivation domain (S1 Appendix and S1 Fig). Our primary interest was to see if the *Cypt* promotor generated comparable levels of full length transcripts from the *Sxr*^*b*^-located *Zfy2/1* fusion gene in X^*Eif2s3y*^Y*^X^*Sxr*^*b*^ males as compared to those from the *Zfy2* transgene in X^*Eif2s3y*,*Zfy2*^Y*^X^ males. Quantification of the *Cypt*-driven transcript bands indicates that this is indeed the case as can be seen from the last two pairs of columns of Fig 6A. It is these transcripts that we contend are supporting the sperm morphogenesis evident in the two models. From Fig 6B it can be seen that the *Zfy2/1* fusion gene present in X*Eif2s3y*Y*X*Sxrb* is expressed at twice the level of the XY*Sxrb* control; furthermore, the *Zfy2* transgene in X^*Eif2s3y*,*Zfy2*^Y*^X^ is also expressed at a substantially higher level than the endogenous *Zfy2* gene of the XY*Sxr*^*b*^ and XY controls. This elevated expression of *Zfy2* is to be expected in the genotypes lacking the Y long arm gene complement because they lack the repressive effect of the estimated 50–100 copies of *Sly* that are involved in the post-meiotic genomic conflict. In whole testis samples from XY males with a targeted knockdown of *Sly* transcripts there is widespread up-regulation of spermatid expressed X and Y genes and this includes *Zfy2* (see [22], Fig 4B). This up-regulation of *Zfy2* is also seen with purified round spermatids (Julie Cocquet, personal communication).

**Fig 7:**
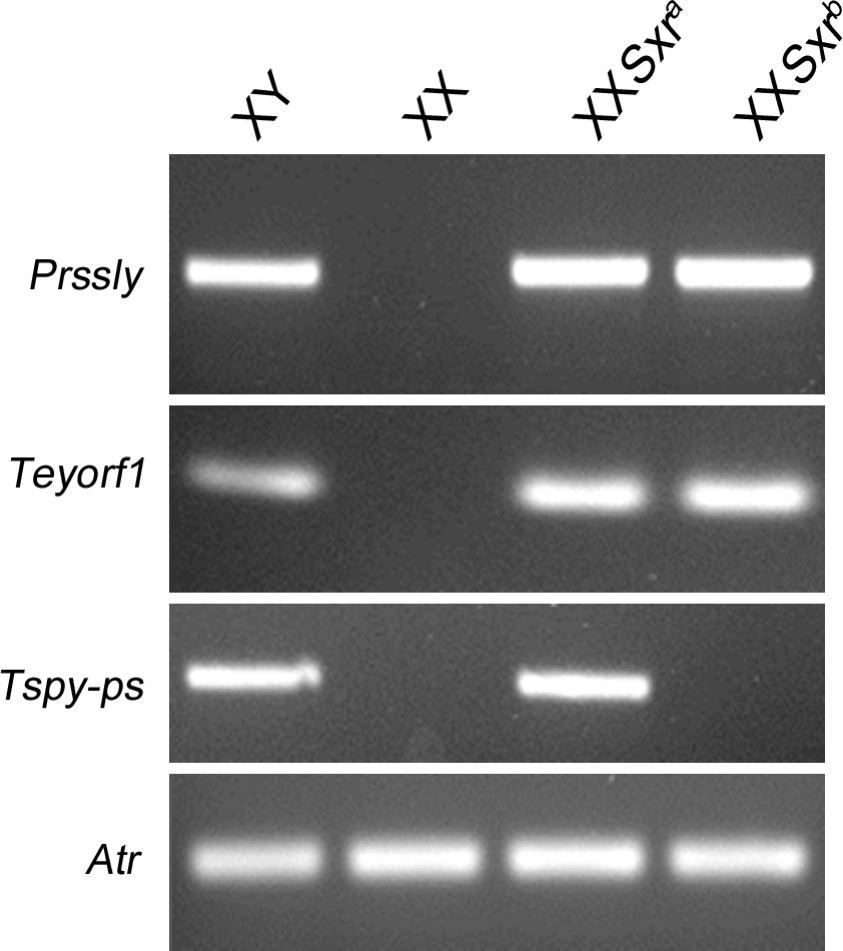
Mapping of *Prssly* and *Teyorf1* to *Sxr*^*a*^ and *Sxr*^*b*^. *Prssly* and *Teyorf1* map to the Yp-derived *Sxr*^*a*^ chromosomal fragment (here attached distal to the PAR of one X of an XX*Sxr*^*a*^ male). As expected from the known breakpoints for the Δ^*Sxr-b*^ deletion, *Prssly* and *Teyorf1* are also present in *Sxr*^*b*^, whereas the *Tspy* pseudogene is absent.

Our finding that sperm morphogenesis in X^*Eif2s3y,Zfy2*^Y*^X^*Sry* males is inferior to that in XY*^X^*Sxr*^*a*^ males points to there being other Yp genes that contribute to sperm morphogenesis. We have generated transgenic lines that express other Yp-located genes (*Ube1y1/Uba1y*, *Smcy/Kdm5d*, *Uty*, *Dby/Ddx3y*, *Usp9y*, *H2al2y*) that may have functions during spermiogenesis. Since our current study was completed two more male germ-line expressed genes, *Prssly* and *Teyorf1*, have been mapped to Yp [36]; these are present in *Sxr*^*a*^ (Fig 7), so they are also candidates for the improvement in sperm morphogenesis in XY*^X^*Sxr*^*a*^ males relative to X^*Eif2s3y,Zfy2*^Y*^X^*Sry* males. However, the breeding required to generate XY*^X^ males with four or more transgenes is now prohibitively expensive. It is therefore encouraging that recent advances in Y gene targeting [37–39] promise simpler and more rapid advances in understanding mouse Yp gene functions than our onerous strategy of Yp transgene addition.

The present findings with respect to the pivotal role of *Zfy2* (but not *Zfy1*) in enabling the transition from randomly orientated round spermatids to spermatids undergoing sperm morphogenesis, are an important addition to our recent findings documenting sometimes overlapping but nevertheless distinct roles for *Zfy1* and *Zfy2* during earlier stages of spermatogenesis. It will be a substantial challenge for the future to document the direct and indirect transcriptional changes triggered by these transcription factors, which underlie their distinct roles.

## Materials and Methods

### Animals

The mice utilized in this study had a limited Y gene complement (see below and Fig 1). Details of their production are provided in Vernet et al., 2014 [14]. All males were examined at 6 weeks of age.

1. X^*Eif2s3y*^O*Sry* (abbreviated as X^*E*^O*Sry*) males carry an X-located transgene encoding the spermatogonial proliferation factor *Eif2s3y* [40] and an autosomally-located transgene of testis determinant *Sry* [41, 42].
2. X^*Eif2s3y*^Y*^X^*Sry* (abbreviated as X^*E*^Y*^X^*Sry*) males have the same Y gene complement as X^*E*^O*Sry* but carry a minute X chromosome derivative (Y*^X^) with a complete pseudoautosomal region (PAR) [18–20].
3. X^*Eif2s3y*^*Sxr*^b^O (abbreviated as X^*E*^*Sxr*^b^O) males have the X chromosome carrying an *Eif2s3y* transgene [40] together with Tp(Y)1Ct^*Sxr-b*^ attached distal to the X PAR. Tp(Y)1Ct^*Sxr-b*^ is an *Sxr*^a^ derivative with a 1.3 Mb deletion that has removed the majority of the Yp gene complement and created a *Zfy2/1* fusion gene [9, 43].
4. X^*Eif2s3y*^Y*^X^*Sxr*^b^ (abbreviated as X^*E*^Y*^X^*Sxr*^b^) have the same Y gene complement as X^*E*^*Sxr*^b^O but also carry Y*^X^.
5. X*Sxr*^*a*^O have the X chromosome carrying the Yp derivative Tp(Y)1Ct^*Sxr-a*^ [44] attached distal to the X PAR.
6. XY*^X^*Sxr*^a^ males have the same Y gene complement as X*Sxr*^*a*^O but also carry Y*^X^.
7. X^*Eif2s3y,Zfy1*^Y*^X^*Sry* (abbreviated as X^*E,Z1*^Y*^X^Sry) males are genotype 2 males to which a single copy, X chromosome-located, *Zfy1*-*Uba1y* BAC (RP24-327G6) transgene [12] has been added.
8. X^*Eif2s3y,Zfy2*^Y*^X^*Sry* (abbreviated as X^E,Z2^Y*^X^Sry) males are genotype 2 to which a single copy *Zfy2* BAC transgene inserted by cassette mediated exchange (CME) into the *Hprt* locus on the X chromosome [12, 13] has been added.

The mice were fed ad libitum with a standard diet and maintained in a temperature and light-controlled room (22°C, 14h light/10h dark). The protocols for animal handling and treatment procedures were reviewed and approved by the Crick Biological Research Facility Strategic Oversight Committee (BRF-SOC) and Animal Welfare and Ethical Review Body(AWERB), in accordance with the United Kingdom Animal Scientific Procedures Act 1986 (Procedure Project Licence: 80/2186).

### Histology and analysis of sperm head morphology

For histology analysis, part of the testes were fixed in Bouin overnight and then stored in 70% ethanol prior to embedding in paraffin wax, sectioning at 5 μm, and staining with hematoxylin-eosin (H&E) or periodic acid Schiff and hematoxylin (PAS-H). The stages of seminiferous tubules were identified based on the composition of cells near the basal membrane according to the method described by Ahmed [45]. This was necessary because of meiotic and post-meiotic arrests present in males with limited Y gene complement, which prevented staging based on the changes of acrosome and nuclear morphology of spermatids. As a consequence, some tubule stages cannot be distinguished reliably so a range has to be given.

Sperm smears obtained from the testis were silver stained and analyzed as described previously [41].

### Transmission electron microscopy

Mice were perfused, through the left ventricle [46], with ice-cold 2.5% glutaraldehyde fixative diluted in PBS. The testes were dissected, left for 1h in the fixative, and cut into small blocks that were kept at 4°C in the same fixative until embedding. Testes were post-fixed for 1h in 1% osmium tetroxide, stained ‘en bloc’ for 1h with 1% aqueous uranyl acetate, dehydrated with graded alcohol series and embedded in Epon. Ultrathin sections (60nm) were contrasted 7min with uranyl acetate and lead citrate and then examined using JEOL 1200EX electron microscope.

### RT-PCR band quantitation for Fig 6

Band intensity was quantified using ImageJ, background subtracted and intensity normalised to *Lemd1* in each sample. For further details see S1 Appendix, S1 and S2 Figs.

### PCR analysis for Fig 7

Standard PCR on genomic DNA was used to map the Y chromosome genes *Prssly* and *Teyorf1*. The controls are *Tspy-p*s, present in the Sxrb deletion interval, and the autosomal gene *Atr*. For primers and annealing temperatures see S1 Table.

## Supporting Information

S1 Appendix Duplex RT-PCR assays. (DOCX)

S1 Fig Duplex RT-PCR assays for Cypt-driven transcript. **(A)** Genomic structure of the mouse *Zfy1*, *Zfy2* and *Zfy2/1* fusion genes are shown. Exons are represented by boxes (black for *Zfy2* and white for *Zfy1*) and are not to scale. The position of the primers used for amplifying Cypt promotor dependent transcripts with exon 6 is depicted below each gene (see S1 Table for the primer sequence). **(B)** Gel picture of the RT-PCR assays showing amplification *Cypt*-dependent transcripts for XY*Sxr*^*b*^, XY, X^*E*,*Z2*^Y*^X^*Sry* and X^*E*^Y*^X^*Sxr*^*b*^. *Lemd1* is a round spermatids expressed gene used as a control.
(TIF)

S2 Fig RT-PCR analysis of staged mouse testes. Lemd1 transcription begins between 20 and 27 dpp. RT-PCR for *Lemd1* and loading control *Hmbs* were performed separately with standard Taq polymerase and mixed for migration. Primers used are in S1 Table.
(TIF)

S1 Table List of primers used. (DOCX)
