# Supplementary material for: Mouse Y-Encoded Transcription Factor Zfy2 Is Essential for Sperm Head Remodelling and Sperm Tail Development
Source: PLoS One. 2016 Jan 14;11(1):e0145398. doi: 10.1371/journal.pone.0145398 (PMC4713206; doi:10.1371/journal.pone.0145398)
Supplement: S1 Appendix — (DOCX) [file pone.0145398.s001.docx]

**S1 Appendix. Duplex RT-PCR assays.**

Duplex RT-PCR assays were performed on 400 ng polyA+ RNA extracted from testis using a μMACS mRNA isolation kit (Miltenyi Biotec). RNA was converted to cDNA using Expand reverse transcriptase (Roche) and a dT_17_ primer. Resulting cDNAs were diluted 10-fold with water and 1 μl used per 25 ml of PCR reaction. PCR was performed with Q5 polymerase (New England Biolabs) using the recommended temperature cycle with 15 s annealing at 67°C and 30 s elongation at 72°C for 30 cycles. We used 0.5 μl of Q5 polymerase for 100 ml of PCR reaction. Primers for the transcript to be tested and the *Lemd1* transcript were at 500 nM each. *Lemd1* was chosen as a control because like Cypt-*Zfy2*, *Lemd1* begins to be transcribed between 20-27 d*pp* as round spermatids mature (S2 Fig). Products were visualised by migration on 2% or 3% agarose gels. Band intensity was quantified using ImageJ, background subtracted and intensity normalised to *Lemd1* in each sample.
