# Supplementary figures and images for: Mouse Y-Encoded Transcription Factor Zfy2 Is Essential for Sperm Head Remodelling and Sperm Tail Development
Source: PLoS One. 2016 Jan 14;11(1):e0145398. doi: 10.1371/journal.pone.0145398 (PMC4713206; doi:10.1371/journal.pone.0145398)

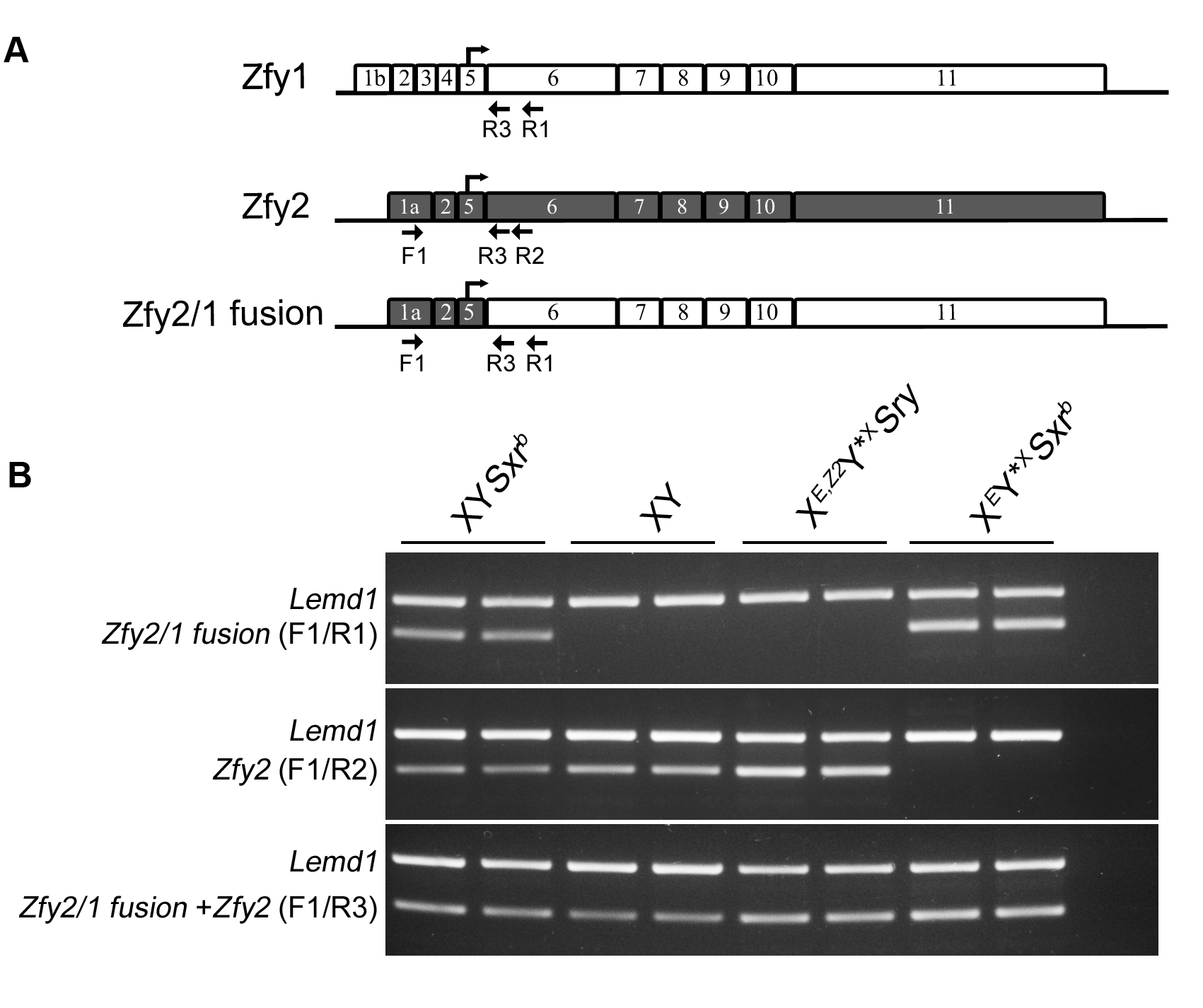

Supplement: S1 Fig — (A) Genomic structure of the mouse Zfy1, Zfy2 and Zfy2/1 fusion genes are shown. Exons are represented by boxes (black for Zfy2 and white for Zfy1) and are not to scale. The position of the primers used for amplifying Cypt promotor dependent transcripts with exon 6 is depicted below each gene (see S1 Table for the primer sequence). (B) Gel picture of the RT-PCR assays showing amplification Cypt-dependent transcripts for XYSxrb, XY, XE,Z2Y*XSry and XEY*XSxrb. Lemd1 is a round spermatids expressed gene used as a control. (TIF) [file pone.0145398.s002.tif]

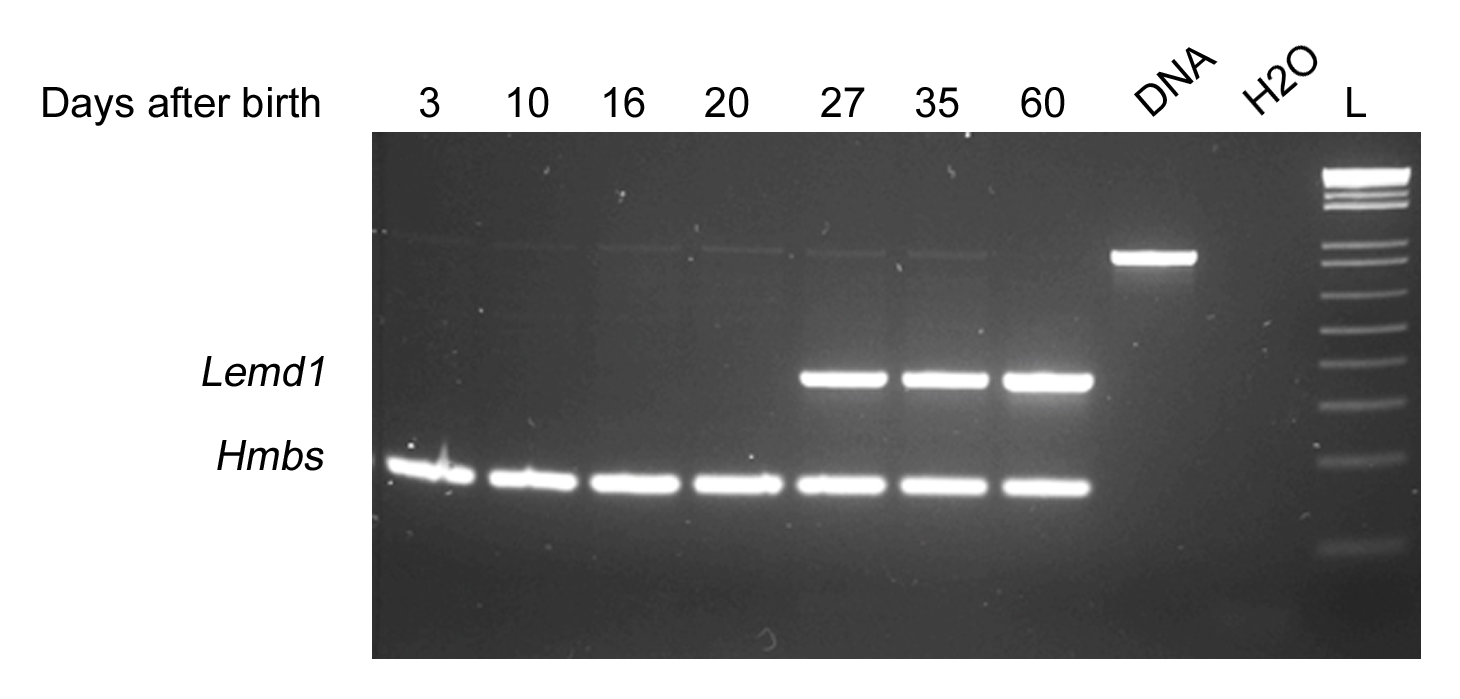

Supplement: S2 Fig — Lemd1 transcription begins between 20 and 27 dpp. RT-PCR for Lemd1 and loading control Hmbs were performed separately with standard Taq polymerase and mixed for migration. Primers used are in S1 Table. (TIF) [file pone.0145398.s003.tif]
