# Supplementary material for: Mouse Y-Encoded Transcription Factor Zfy2 Is Essential for Sperm Head Remodelling and Sperm Tail Development
Source: PLoS One. 2016 Jan 14;11(1):e0145398. doi: 10.1371/journal.pone.0145398 (PMC4713206; doi:10.1371/journal.pone.0145398)
Supplement: S1 Table — (DOCX) [file pone.0145398.s004.docx]

**S1 Table.** **List of primers used.**

| Gene | Primer ID | Primer sequence | Annealing temperature  Q=Q5 pol PCR | Amplicon  size | |  |  |  |
| --- | --- | --- | --- | --- | --- | --- | --- | --- |
| **Primers for duplex PCR** | | | | |  | |  | **Primers for duplex PCR** |
| *Zfy2/1* | F1 | TGTTGTGGTTCTCGTAGCAGA | Q67°C | 238 bp | |  |  |  |
|  | R1 | GTTTCTTGTACTTCCACAACAATCTGG |  |  |  |  |  |  |
|  |  |  |  |  | |  |  |  |
| *Zfy2* | F1 | TGTTGTGGTTCTCGTAGCAGA | Q67°C | 238 bp | |  |  |  |
|  | R2 | TGTACTGCATCAGCTCCTATTCC |  |  |  |  |  |  |
|  |  |  |  |  | |  |  |  |
| *Zfy2 and Zfy2/1* | F1 | TGTTGTGGTTCTCGTAGCAGA | Q67°C | 203 bp | |  |  |  |
|  | R3 | TGTACTGCATCAGCTCCTATTCC |  |  | |  |  |  |
|  |  |  |  |  | |  |  |  |
| **Primers for loading control** | | | | |  | |  | **Primers for loading control** |
| *Hmbs* | HmbsF | CTGAAAGCCTTGTACCCTGG | 60°C/ Q67°C | 170 bp | |  |  |  |
|  | HmbsR | GAGTGAACGACCAGGTCCAC |  |  |  |  |  |  |
| **Primers for spermatid control** | | | | |  | |  | **Primers for spermatid control** |
| *Lemd1* | Lemd1F | GATTGCATAGACTTCAGACAACC | 60°C/ Q67°C | 358 bp | |  |  |  |
|  | Lemd1R | GCCTCAAGTCTCTTCTTGCC |  |  |  |  |  |  |
| **Primers for *Prssly* and *Teyorf1* mapping** | | | | |  | |  | **Primers for *Prssly* and *Teyorf1* mapping** |
| *Prssly* | PrsslyF | GAAGTCCTGGCATCCTGCAA | 60°C | 154 bp | |  |  |  |
|  | PrsslyR | AATGGTGCCCCAAGATCACC |  |  |  |  |  |  |
| *Teyorf1* | Teyorf1F | TGCTGGATCAGAGCCCCATA | 60°C | 117 bp | |  |  |  |
|  | Teyorf1R | CACCACATGGTTCCAGCTCA |  |  |  |  |  |  |
| *Tspy-ps* | TspyF | GTGGAAGAGTACAACACTGG | 60°C | 340 bp | |  |  |  |
|  | TspyR | CTCAGCAATCCTGTTGGAGC |  |  |  |  |  |  |
| *Atr* | AtrF | GGGATGTTTACAGCCAGCTC | 60°C | 142 bp | |  |  |  |
|  | AtrR | AGCCGATTTGCCACAGTAAC |  |  |  |  |  |  |
